# Endocrine Hypertension: The Urgent Need for Greater Global Awareness

**DOI:** 10.17925/EE.2023.19.2.11

**Published:** 2023-10-20

**Authors:** Cornelius J Fernandez, Lakshmi Nagendra, Mohammed Alkhalifah, Joseph M Pappachan

**Affiliations:** 1. Department of Endocrinology and Metabolism, Pilgrim Hospital, United Lincolnshire Hospitals NHS Trust, Boston, UK; 2. Department of Endocrinology, JSS Medical College, JSS Academy of Higher Education and Research, Mysore, India; 3. Department of Endocrinology and Metabolism, Lancashire Teaching Hospitals NHS Trust, Preston, UK; 4. Department of Family Medicine & Diabetes, King Saud University Medical City, Riyad, Saudi Arabia; 5. Faculty of Science, Manchester Metropolitan University, Manchester, UK; 6. Faculty of Biology, Medicine and Health, The University of Manchester, Manchester, UK

**Keywords:** Endocrine hypertension, monogenic hypertension, adrenal disorders, primary aldosteronism (PA), pheochromocytoma, Cushing's syndrome

## Abstract

Hypertension affects about 1.28 billion adults globally, and significantly increases the risk of chronic morbidity and mortality among sufferers. About 15% of these individuals have secondary hypertension, the majority of whom have dysfunction of one or more endocrine systems as the cause of hypertension. Although adrenal disorders are often identified as the cause of endocrine hypertension, extra-adrenal disease and pituitary disorders also can cause the disease. Timely diagnosis is of paramount importance, because of the potential for a surgical cure or optimal disease control with pharmacotherapy to prevent hypertensive complications. Even with its relatively high prevalence compared with many other chronic illnesses, the diagnosis of endocrine hypertension is often delayed or never made because of poor awareness about the disease among physicians. This review attempts to provide an overview of the disease, with some practical aspects of diagnosis and management of a few of the important disorders causing endocrine hypertension.

Hypertension affects up to 40% of the adult population worldwide,^1^ and according to the World Health Organization's 2021 estimates, globally 1.28 billion adults between 18 and 79 years are affected.^2^ Of these, 85% have essential hypertension^3^ and the remainder have secondary hypertension, which is potentially curable with timely diagnosis and treatment.^3^ The prevalence of secondary hypertension is around 50% in hypertensive children,^4^ and 30% in young adults under the age of 40 years^4,5^ indicating that children and young adults should undergo secondary hypertension workup, even outside the resistant hypertension range.

Secondary hypertension often presents as resistant hypertension, which is defined as blood pressure that remains above 140/90 mmHg despite the use of three antihypertensive medications (of which one is a diuretic) of different classes at the maximum tolerated doses.^6^ The prevalence of secondary hypertension is only 5–15% in the non-s elected hypertensive population, whereas it can be around 30% in patients with resistant hypertension.^7^ Hence, working up patients with resistant hypertension is crucial to identifying a potential secondary cause.^8^

Endocrine causes of secondary hypertension (endocrine hypertension) have been increasingly identified in recent years in parallel with improvement in our diagnostic armamentarium. They are now among the most frequent causes of secondary hypertension, the other common causes of secondary hypertension being renal parenchymal or renovascular disease, coarctation of the aorta and obstructive sleep apnoea (OSA). With the recognition of the increased prevalence of primary aldosteronism, they could represent up to 20% of patients with hypertension.^9^ The main causes of endocrine hypertension are summarized in *Table 1*.^10^ In this article, we will mainly focus on three important causes of endocrine hypertension (primary aldosteronism, Cushing's syndrome and pheochromocytoma), as the more common causes such as thyroid and parathyroid diseases, are already familiar to most readers.

Investigating the cause of endocrine hypertension can be highly rewarding for both patients and physicians, as identifying a treatable endocrine disease can greatly improve the outcome, although this requires a high index of suspicion. However, the diagnosis is often delayed, or even never made due to inadequate awareness among physicians. Moreover, the clinical presentation of some of these disorders can be vague, mimicking other systemic diseases.^11^

## Monogenic hypertension

**Figure 1:**
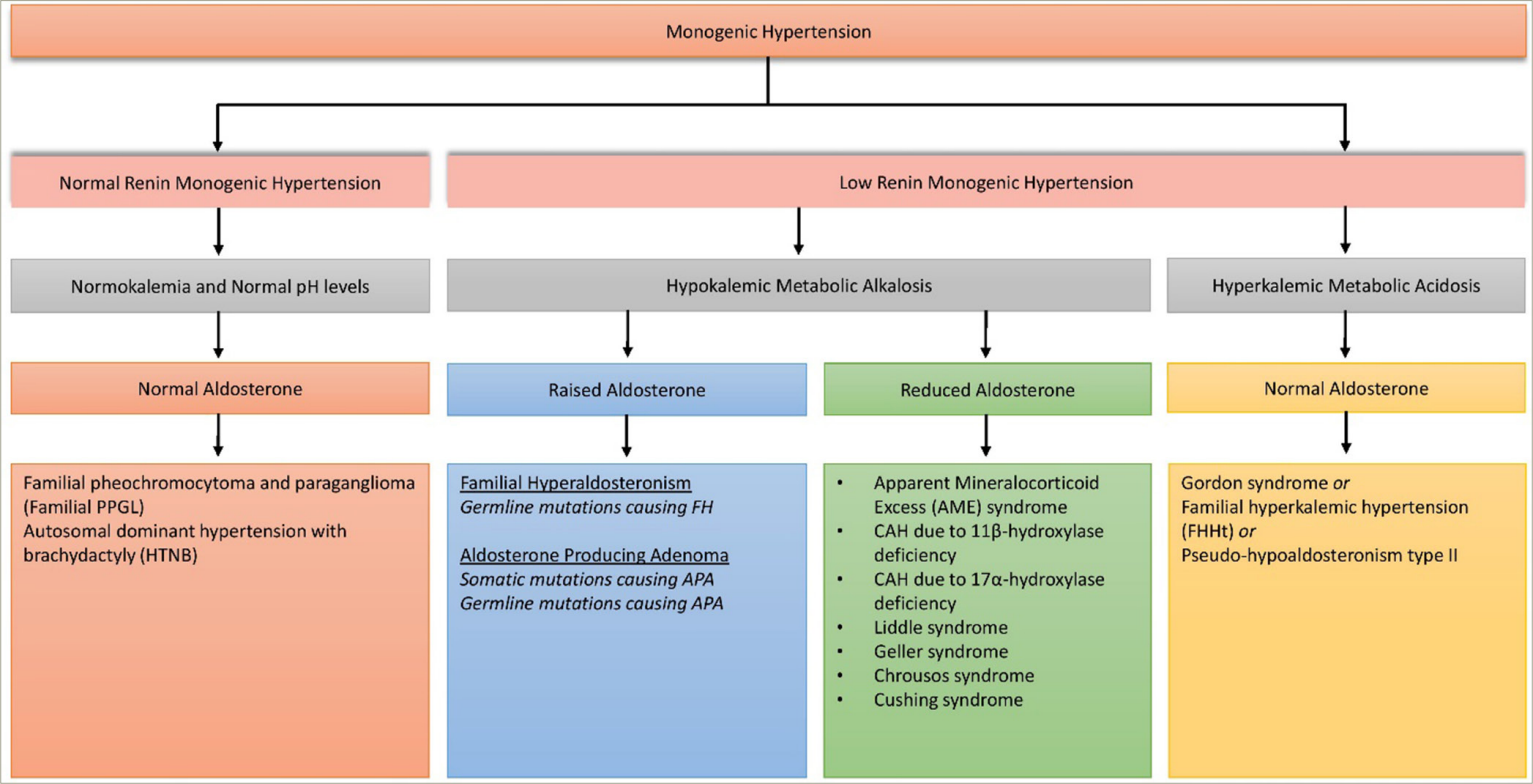
The classification of monogenic hypertension

Essential hypertension is a polygenic trait and over 900 genetic variants have been known to contribute to its development, together with several environmental factors. In contrast, many causes of secondary hypertension are monogenic, that is, they are associated with single gene alterations with a Mendelian pattern of inheritance.^12^ An overview of monogenic hypertension is given in *Figure 1*. The typical presentation of monogenic hypertension is childhood-onset, severe or resistant hypertension and a positive family history. However, due to incomplete penetrance, some genetic carriers show milder hypertension, or even normotension. Moreover, typical electrolyte abnormalities may not be present in all carriers. Accompanying syndromic features, including brachydactyly, should trigger the performance of genetic tests. Measurement of renin and aldosterone levels coupled with genetic testing is indicated if monogenic hypertension is suspected.^12^

**Table 1:** The main causes of endocrine hypertension

Endocrine glands involved	Mechanism and diagnosis of endocrine hypertension
Adrenal	Mineralocorticoid-mediated hypertension
	Primary aldosteronism Familial hyperaldosteronism Apparent mineralocorticoid excess syndrome Congenital adrenal hyperplasia due to 11β-hydroxylase deficiency CAH due to 17α-hydroxylase deficiency Geller syndrome Liddle syndrome Deoxycorticosterone-producing tumour Glucocorticoid-mediated hypertension Adrenal Cushing's syndrome Chrousos syndrome Catecholamine-mediated hypertension Pheochromocytoma Paraganglioma
Pituitary	Acromegaly Cushing's disease
Thyroid	Hypothyroidism Hyperthyroidism
Parathyroid	Primary hyperparathyroidism

## Primary aldosteronism

PA is the most common cause of curable endocrine hypertension, with an estimated prevalence of >10% among hypertensive patients and 20% among those with resistant hypertension.^13^ Biochemically overt PA diagnosed with urinary aldosterone levels greater than 12 μg/24 h can be seen in 15.7%, 21.6% and 22.0% of patients with stage 1 hypertension, stage 2 hypertension and resistant hypertension, respectively.^14^ In PA, excessive aldosterone production inappropriate to the sodium levels leads to increased sodium reabsorption, plasma volume expansion and, as a result, hypertension.^13^ This is associated with increased potassium and hydrogen ion excretion and may lead to hypokalemic metabolic alkalosis. PA can be caused by bilateral adrenal hyperplasia (BAH: 60%), aldosteroneproducing adenoma or Conn's syndrome (APA: 35%), unilateral adrenal hyperplasia (2%) and, rarely, adrenocortical carcinoma (ACC <1%), ectopic APA (<1%) and various subtypes of familial hyperaldosteronism including FH-I (1.2–6.0%), FH-I I (<1%), FH-I II (rare), FH-I V (rare) and PASNA (rare).^15^ The differentiation between APA and BAH is vital, as APA can be cured surgically, whereas BAH is treated medically, and this process depends on the availability of adrenal vein sampling (AVS).

The PA diagnostic pathway involves screening, confirmatory and subtyping tests. Early detection and specific therapy are crucial as undiagnosed/untreated PA is associated with raised renal, cardiovascular, cerebrovascular, and metabolic morbidity and mortality through direct target organ damage independent of hypertension.^16^ Inappropriate mineralocorticoid receptor (MR) activation can cause endothelial dysfunction, myocardial fibrosis and microalbuminuria.^13^ Glucose metabolism is impaired, and the increased prevalence of diabetes and metabolic syndrome further increases cardiovascular risk.^16^ Though early detection of PA is vital, screening for PA is reported in only 8–9% of those with hypertension, and general practitioners demonstrate a surprisingly little knowledge about PA and its diagnostic guidelines.^17^ PA remains largely under-recognized due to underestimation of its prevalence, the complexity of the diagnostic tests, and the lack of familiarity with the interpretation of the diagnostic test results. *Table 2* gives a summary of hypertensive patients who should undergo screening for PA.

*APA = aldosterone producing adenoma; CAH = congenital adrenal hyperplasia; FH = familial hyperaldosteronism.*

**Table 2:** Hypertensive patients with a high pre-test probability of primary aldosteronism for screening with aldosterone-to-renin ratio^18,19^

Resistant Hypertension: BP ≥140/90 mmHg despite therapy with three medications
Hypertensives with moderate or severe disease
Hypertensives with spontaneous/diuretic-induced hypokalaemia
Hypertensives with adrenal incidentaloma
Hypertensives with obstructive sleep apnoea
Hypertension with a family history of early onset hypertension or CVA at <40 years of age
All hypertensive first-degree relatives of patients with PA
Unexplained atrial fibrillation^19^
Greater target organ damage (microalbuminuria, CKD, LVH, diastolic dysfunction) than expected based on blood pressure values^19^

*BP = blood pressure; CKD = chronic kidney disease; CVA = cerebrovascular accident; LVH = left ventricular hypertrophy.*

Aldosterone-to-renin ratio (ARR) is the most simple, reliable and reproducible PA screening test. It should be measured more than once before planning a confirmatory test. A normal or raised plasma aldosterone concentration (PAC) with a low or suppressed plasma renin activity (PRA) or direct renin concentration (DRC) gives rise to an elevated ARR.^20^ ARR has high sensitivity but low specificity. It is more sensitive than raised aldosterone and more specific than suppressed renin when taken in isolation. The ARR threshold to diagnose PA is dependent on the PAC and PRA or DRC assays used. PAC is conventionally measured using immunoassays (enzyme immunoassay [EIA] or chemiluminescence immunoassay [CLIA]). The newer liquid chromatography-mass spectrometry (LC-MS/MS) technique has much improved specificity, accuracy and reproducibility. The PAC measured with LC-MS/MS is much lower in comparison to that with the immunoassay. Hence, the LC-MS/MS avoids falsely high PAC levels at the lower limit of the reference range, which is very useful during confirmative tests. PRA also uses immunoassays or LC-MS/MS. DRC uses automated immunometric assays, which are faster, cheaper and less labour intensive than PRA, and it allows sample handling at room temperature. However, they are less reliable and reproducible, especially at the lower limit of the reference range. PRA therefore remains the preferred approach.^20^

The ARR is highly determined by renin. In the presence of very low renin values, the ARR may be elevated even when the PAC values are very low and not compatible with PA. To avoid a falsely high ARR, the lowest level of renin is fixed at 0.2 ng/ml/h for PRA and 2 mUI/l for DRC.^19^ This is especially important in people with low renin levels, including the elderly and people of African origin.^21^ Some guidelines use a minimum PAC threshold of >277 pmol/L (>10 ng/dL)^22^ within the screening criteria to avoid these false high ARR values. Others use a higher PAC threshold of >416 pmol/L (>15 ng/dL).^19^ Others still use a lower PAC threshold of >162 pmol/L (5.8 ng/dL), the normal suppression after a seated saline suppression test, when measured by LC-MS/MS.^20^ Including a “PAC threshold” has been shown to increase the specificity but reduce the sensitivity of the ARR.^23^ Therefore, a PAC threshold is not widely accepted in PA screening. However, the PAC should be above the cut-off for the subsequent confirmatory test.

As people of African origin are very sensitive to aldosterone, PA can manifest with only mild PAC elevations and, therefore, different ARR and PAC cut-offs might be used.^19^ Moreover, normal PAC levels seen in PA patients should be considered as *inappropriately normal* in the face of suppressed renin levels which, in subjects without PA, should have caused low PAC levels.^20^ Finally, the sexually dimorphic relationship between ARR and blood pressure in young adults indicates that sexspecific ARR cut-offs are needed in young adults.^24^

**Table 3:** Preparations for an ideal aldosterone-to-renin ratio test^18–20^

ARR has high sensitivity if measured in an upright posture in the morning. The patient should be up (sitting, standing or walking) for at least 2 h and seating for 5–15 minutes
Correct hypokalaemia and aim for serum potassium >4 mmol/L to avoid falsenegative ARR
It is vital not to restrict sodium intake (<75 mEq/day) in the days before ARR to avoid false-negative ARR. Also, avoid sodium loading (>300 mEq/day) to minimize false-positive ARR
Withdraw drugs with marked effect on ARR for at least 4 weeks, including: potassium-sparing diuretics (spironolactone, eplerenone, amiloride and triamterene), potassium-wasting diuretics, and products from liquorice root (confectionary liquorice, chewing tobacco)
If ARR is non-diagnostic, withdraw even the drugs with lesser effect on ARR for at least 2 weeks: β-blockers, clonidine, α-methyldopa, and NSAIDs
Antihypertensive drugs with the least effect on ARR (hydralazine, moxonidine, α1-selective adrenergic blockers, or slow-release verapamil) are recommended for BP control if needed
β-blockers, clonidine, α-methyldopa, and NSAIDs can cause false-positives ARR
ACEi, ARBs, diuretics (all) and dihydropyridine CCBs can cause false-negative ARR
ARR that is done while on interfering drugs can still be important
Normal ARR while on β-blockers would make PA unlikely; raised ARR with suppressed renin while on ACEi, ARBs or diuretics (not β-blocker) would make PA very likely
Premenopausal women should have the ARR test in the follicular phase, as the luteal phase or OCP/HRT affects DRC ARR with DRC, but not PRA is associated with false-positive ARR

*ACEi = angiotensin convertase enzyme inhibitor; ARB = angiotensin II receptor blocker; ARR = aldosterone-to-renin ratio; BP = blood pressure; CCB = calcium channel blocker; DRC = direct renin concentration; HRT = hormone replacement therapy; NSAID = nonsteroidal anti-inflammatory drug; OCP = oral contraceptive pill; PA = primary aldosteronism; PRA = plasma renin activity.*

*Table 3* summarizes the preparations for an ideal ARR test. The natural history of PA starts with normotension and renin suppression, progressing to stage 2 or 3 and/or resistant hypertension.^25,26^ Some authors suggest considering ARR in all hypertensives at the time of diagnosis before commencing antihypertensive drugs.^27^ This would avoid confounding drug effects and permit the earlier introduction of specific therapy. Restricting screening to hypertension with hypokalaemia is inappropriate as this would miss up to 80% of PA.^27^ OSA is common in PA, resulting from PA-induced fluid retention causing upper airway soft tissue oedema. PA therapy, either with mineralocorticoid receptor antagonists (MRAs) or adrenalectomy, achieves a significant reduction in neck circumference and apnoea-hypopnoea index.^28^ Similarly, PA is common in OSA due to the hypoxia-mediated rise in plasma endothelin-1 levels increasing the aldosterone secretion.^29^

As the renin and PAC are measured using varied assays, and the results are available in different units from different laboratories, it is not possible to establish a rigid diagnostic threshold for ARR. The performance of ARR, especially the sensitivity, differed markedly based on the study population and diagnostic threshold. Some recommend concentrating on the actual PAC and renin values that generated the ARR rather than the ARR itself. When renin is measured as PRA, one of the best ARR threshold values is ≥30 with PAC in ng/dL and PRA in ng/mL/h with a PAC threshold of >10. This is equivalent to an ARR threshold of ≥830 with PAC in pmol/L and PRA in ng/mL/h with a PAC threshold of >277.^30^ Selecting an ARR threshold of ≥40 and a PAC threshold >15 ng/dl would be less sensitive but more specific.^30^ When renin is measured as DRC, one of the best ARR threshold values is ≥2.4 when PAC is in ng/dL and DRC is in mU/L or ≥70 when PAC is in pmol/L and DRC is in mU/L.^31^

In an Italian study, where renin is measured as DRC using CLIA, an ARR threshold of 2.06 ng/dl per mU/L, which is equivalent to 20.6 ng/mU, provided a sensitivity of 92%, a specificity of 92% and an overall accuracy of 97.4%.^32^ The same study observed that, when renin is measured as PRA using RIA, an ARR threshold of 38.7 ng/dL per ng/mL/h provided a sensitivity of 80% and a specificity of 92%. In a similar Spanish study, where renin is measured as DRC using CLIA, an ARR threshold of 2.3 ng/ dl per mU/L (equivalent to 23 ng/mU) provided a sensitivity of 100% and a specificity of 90.9%.^33^ When renin is measured as PRA using RIA, an ARR threshold of 48.9 ng/dL per ng/mL/h provided a sensitivity of 100% and a specificity of 93.6%.

Significant within-individual variability in PAC (22–25%), DRC (41–42%) and ARR occurs in a large volume of patients investigated for PA.^31^ Nearly 38% had at least one ARR below the threshold, indicating that ARR needs to be done a minimum of twice before PA is excluded or confirmed. As per the Endocrine Society's recommendations, those who have a persistently raised ARR should undergo one or more of the confirmatory tests, the exception being those with hypokalaemia, and those with suppressed renin below the detection limits plus with PAC >20 ng/dL (550 pmol/L).^18^ Additionally, markedly raised ARR (e.g. >45 ng/mU) increases the probability of PA, with significantly low false-positive rates.^34^ Hence, among patients with markedly raised ARR, those who are surgical candidates can undergo adrenal venous sampling (AVS) even without doing confirmatory tests, reflecting the quantitative importance of ARR.

The higher the ARR and the PAC threshold, the greater the number of patients established to be truly positive for PA. At an ARR >30 ng/dL per ng/ml/h and a PAC >10 ng/dL, 42.7% of the ARR were true positives.^30^ At an ARR >20 ng/dL per ng/ml/h with PAC ≥10 ng/Dl, 33.3% were true positives.^35^ Finally, at an ARR >20 ng/dL per ng/ml/h and PAC >15 ng/dL, only 28.6% were true positives.^36^ ARR, as a screening test, has a better discriminating ability in patients with hypokalaemia. Hence, ARR alone may be sufficient for diagnosis in these patients and confirmatory tests can be skipped.^37^ On the other hand, among normokalaemic hypertensives, there is a considerable overlap in ARR between those with PA and essential hypertension. Hence, caution should be exercised before considering skipping confirmatory tests in this setting.^37^

Confirmation of PA depends on the demonstration of autonomous aldosterone secretion in the presence of dynamic procedures that are designed to suppress aldosterone levels, so that false-positive diagnoses and thereby inappropriate adrenal venous sampling and surgery are avoided. The available confirmatory tests include the fludrocortisone suppression test (FST), saline infusion test (SIT), and oral sodium loading test (OSLT), all of which are meant to expand the plasma volume and suppress renin. The remaining available confirmation tests include the captopril challenge test (CCT), which is meant to suppress the aldosterone through inhibition of angiotensin-converting enzyme, losartan suppression test and furosemide upright test.^30^

Currently, there is no universal gold standard confirmatory test. Fludrocortisone suppression test (FST), though considered to have maximum reliability, requires hospital admission for 5 days and is laborious, hence it is rarely done nowadays. Originally, SIT was done with the patient in the recumbent position. However, performing SIT in the seated position results in marked improvement in sensitivity, while retaining the specificity. The confirmatory tests that are meant to expand the plasma volume are contraindicated in severe hypertension, and in renal or cardiac dysfunction.^13^ In these patients, though the captopril challenge test can be used, this test has a low discriminatory power; moreover, it can cause symptomatic hypotension. It is therefore sensible to directly proceed to subtyping (imaging or AVS) or to consider a trial of medical treatment.^30^ A recent systematic review and meta-analysis that evaluated the performance of various confirmatory tests observed that the routine use of these tests should be reconsidered due to large variations in the way they were performed, interpreted and verified.^38^

This should be followed by PA subtyping, initially with either a triple-p hase adrenal computed tomography (CT) or adrenal magnetic resonance imaging (MRI), followed by AVS. Adrenal CT can identify unilateral or bilateral adrenal adenomas, micronodular/macronodular adrenal hyperplasia or, rarely unilateral adrenocortical carcinoma.^30^ Adrenal CT will not be able to comment on the nodules’ functional status. As adrenal adenomas are very common in the elderly population, the accuracy of adrenal CT in elderly for subtyping is low. On the contrary, due to adrenal adenomas being uncommon in the younger population, detecting a unilateral adrenal adenoma above 10 mm in a younger patient with PA is high likely to indicate an ipsilateral disease. Adrenal MRI is marginally more sensitive, but less specific in comparison with adrenal CT due to lower spatial resolution. Moreover, it is subject to motion artifacts.

Although AVS is the gold standard investigation for subtyping, it is a challenging technique as it is not widely available, is poorly standardized, and must be performed only by an experienced interventional radiologist.^30^ In experienced hands, the success rate can be above 95% where it can successfully localize the source of aldosterone overproduction as unilateral or bilateral.^30^ During AVS, both adrenal veins are catheterized simultaneously or sequentially and blood samples for aldosterone and cortisol are collected from both adrenal veins and from the inferior vena cava (IVC). Adrenal vein to IVC cortisol ratio (C_AV_/C_IVC_) >3.0 from right and left adrenal veins indicates successful adrenal vein catheterization on either side. Dividing the right and left adrenal vein aldosterone levels by their respective cortisol level provides cortisol-corrected aldosterone ratios (ACR).^20,30^ Then, dividing higher ACR to lower ACR known as lateralization index (ACR_dominant_/ACR_contralateral_) >4.0 indicates unilateral aldosterone excess. Together with this, dividing lower ACR to that from IVC (ACR_contralateral_/ACR_IVC_) provides the contralateral suppression index. If this is <0.5 it confirms unilateral aldosterone excess. Nearly 50% of centres use synthetic adrenocorticotropin (ACTH) infusion during AVS to lessen the stress-induced aldosterone fluctuations, to augment the adrenal vein to IVC cortisol gradient, and to enhance the secretion of aldosterone from aldosterone-producing adenomas.^20^

CT adrenal alone is inadequate for surgical referral, as concordance between CT-based and AVS-based strategies was only about 50%.^39^ AVS uncovered unilateral disease in 22% of CT-negative patients, whereas CT identified a unilateral mass in 25% of patients with bilateral or contralateral disease at AVS. Thus, if a referral is made based only on CT, one-fifth of patients would have been denied surgery, and few others would have undergone needless surgery.

The gold standard therapy for unilateral APA is unilateral adrenalectomy, where the laparoscopic approach is preferred.^20^ During the first postoperative day, blood pressure and potassium must be rigorously checked. Discontinuation of MRA and potassium supplement should be considered to prevent hypotension/hypokalaemia.^20^ Medical therapy of PA is recommended in bilateral adrenal hyperplasia, bilateral APA, unilateral APA with aldosterone-producing cell clusters (APCCs), familial hyperaldosteronism, and in patients with unilateral APA who refuses surgery or in whom surgery is contraindicated.^20^ FH-I (GRA or glucocorticoid-remediable aldosteronism) is treated with low dose glucocorticoids with the least mineralocorticoid activity (ideally dexamethasone 0.125–0.25 mg nocte). The side effects of spironolactone can be lessened by low doses or with co-administered epithelial sodium channel (ENaC) inhibitors.

**Figure 2:**
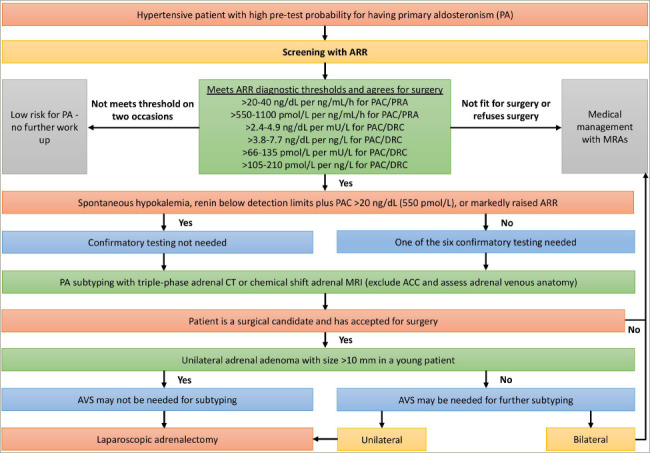
Diagnostic and management approach to a patient with suspected primary aldosteronism

*ACC = adrenocortical carcinoma; ARR = aldosterone renin ratio; AVS = adrenal venous sampling; CT = computed tomography; PAC = plasma aldosterone concentration; PRA = plasma renin activity; MRA = mineralocorticoid antagonist; DRC = direct renin concentration.*

PA is a major public health issue that needs to be addressed. Based on the 24 h urinary aldosterone excretion (UEA) test as a measure of aldosterone status, the prevalence of PA seems to be 3–5 times greater than the currently recognized number of 5–10% of hypertensives.^39^ Patients with inadequately treated PA have a threefold greater cardiovascular risk than that of matched essential hypertensives. Finally, <1% of hypertensives are ever screened for PA, increasing the risks associated with untreated PA on top of those for hypertension per se.^39^ A diagnostic and management approach to a hypertensive patient with suspected PA is shown in *Figure 2*.

## Cushing's syndrome

Cushing's syndrome is characterized by a wide range of clinical manifestations that result from chronic exposure to elevated glucocorticoids of either exogenous or endogenous origin. Patients with Cushing's syndrome often have central fat accumulation, hypertension, diabetes mellitus, fatigue, menstrual abnormalities, hirsutism, decreased libido, dorsocervical fat pad, supraclavicular fullness, mood disturbances and peripheral oedema. However, the above-mentioned clinical picture is often nonspecific and may be seen commonly in the general population (Cushingoid appearance) because of the increase in global obesity prevalence. Clinical features with a high specificity for CS include facial plethora, spontaneous bruising, broad violaceous striae (>1 cm), unexplained osteoporosis, and proximal myopathy.^40,41^ On laboratory evaluation, these patients may exhibit hypokalaemia (mineralocorticoid receptor activation from cortisol excess), hyperglycemia (especially post-prandial hyperglycaemia),^42^ hyperandrogenism, secondary hyperparathyroidism, leucocytosis and raised liver enzymes.

Untreated CS is associated with infections, cardiovascular complications, malignancies and increased mortality. Patients with CS have a four-to fivefold higher risk of developing cardiovascular disease in comparison with the general population, and the risk factors include hypertension, dyslipidaemia, diabetes mellitus, obesity and hypercoagulability.^41^ Although early treatment can achieve a significant reduction in the mortality associated with CS, it cannot fully mitigate the risk. This could partly be due to recurrent or residual disease in some patients. However, the risk continues even in those with apparently successful treatment. The risk of long-term excess mortality associated with CS seems to vary depending on the aetiology of CS, and most patients with ectopic CS have a poorer prognosis, followed by treated Cushing's disease in comparison to cases of treated adrenal CS.^40,41^

CS is an uncommon cause of secondary hypertension. In general outpatient clinics, the prevalence of CS is around 0.5% in the hypertensive population and <1% in patients with resistant hypertension.^40^ Only hypertensive patients with other clinical features of CS should undergo screening tests for CS.^40^ Endogenous CS can either be ACTH-dependent (80% of all cases) or ACTH-independent (20%). ACTH-dependent CS includes ACTH-secreting pituitary adenoma (known as Cushing disease; CD), and ectopic ACTH or CRH (corticotrophin-releasing hormone) producing tumour (known as ectopic ACTH secretion or EAS). On the other hand, ACTH-independent CS includes adrenal glucocorticoid excess (adrenal CS).

Endogenous CS is a rare disease with an incidence of 1.2 to 2.4 per million/year and a prevalence of 40 per million population.^43^ On the contrary, CS due to exogenous glucocorticoid exposure is much more common. Hence, ruling out exogenous cortisol (injection, oral, inhaler or topical) is the first step in all patients in whom CS is suspected. This should be followed by confirmation of the cortisol excess with one of the following tests: either at least two measurements of 24 h urinary free cortisol excretion (UFC), two late-night salivary cortisol measurements (LNSC), or an overnight dexamethasone suppression testing (ODST).^40,41^ These screening tests utilize three cardinal features of endogenous CS, and these include the increased cortisol production (UFC), the loss of normal circadian rhythm in cortisol production (LNSC), and the failure to suppress the autonomous cortisol secretion by the exogenously administered glucocorticoids (DST).^41^ If adrenal CS is suspected, the use of either ODST or LNSC is preferred as the initial screening test. Patients with an abnormal initial test require at least one additional positive test to confirm the diagnosis. Other tests such as the dexamethasone-CRH test may further help in the process of confirmation.

UFC is a measure of free cortisol that is not bound to the cortisol-binding globulin (CBG). Urine should be collected at least twice, and the urine cortisol should be measured together with the urine creatinine and 24 h urine volume to ensure adequacy of urine collection. UFC may be inaccurate in patients with chronic kidney disease. Loss of normal circadian rhythm in cortisol production in patients with CS results in failure to reach a nadir in cortisol levels at late night, thereby having raised late-night salivary cortisol (LNSC) values. LSNC should be measured on multiple occasions at bedtime or midnight. LSNC may be inaccurate in those who smoke, shift workers, or those with oral bleeding and infection. In healthy subjects, 1 mg of dexamethasone taken at 11 p.m. should inhibit ACTH and cortisol production (with cortisol values <1.8 ug/ dL or 50 nmol/L). Dexamethasone levels can be measured to ensure that the patient has performed the test correctly, or that the patient does not have either one of the following: impaired dexamethasone absorption, raised dexamethasone metabolism (caused by drugs like carbamazepine and phenytoin, or lowered dexamethasone clearance (renal or hepatic insufficiency). ODST is considered the first-l ine screening test to look for CS in patients with an adrenal incidentaloma. This test may be inaccurate in those with alterations in CBG and albumin, including those on estrogen. All three screening tests for CS lack specificity, though they all exhibit high sensitivity.

The next step in the diagnosis of CS is to determine whether the disease is ACTH-dependent or ACTH-independent. ACTH <10 pg/mL or ng/L indicates ACTH-independent hypercortisolism (suggesting adrenal CS), though the level can be 10–20 pg/mL in patients with adrenal CS.^40^ Elevated dehydroepiandrosterone sulfate (DHEA-S) associated with low ACTH should raise suspicion of adrenal carcinoma. Patients with ACTH-independent hypercortisolism should be investigated with dedicated CT/MRI of adrenal glands, followed by consideration of adrenal venous sampling in potential surgical candidates.^41^

ACTH >20 pg/mL or ng/L indicates ACTH-dependent hypercortisolism.^40^ A single test can’t differentiate CD from EAS. Imaging, dynamic testing, and clinical features in combination are recommended in the determination of the source of ACTH overproduction. As pituitary adenoma is the commonest cause of ACTH-dependent CS, a dedicated pituitary MRI should be done. An observation of an adenoma >10 mm supports the diagnosis of CD. However, around 12% of EAS patients can also exhibit abnormal pituitary imaging. Moreover, normal pituitary imaging can’t rule out the pituitary origin of CS as up to 40% of corticotroph adenomas are unseen on MRI.^44^

Inferior petrosal sinus sampling (IPSS) is the investigation to look for the source of ACTH in cases with pituitary adenomas <6–9 mm or in those with no adenoma/equivocal pituitary imaging.^40^ IPSS is based on the measurement of ACTH from both the right and left petrosal sinuses in comparison to the peripheral vein. A central to peripheral ACTH gradient >2 without or >3 with CRH or desmopressin stimulation is consistent with a diagnosis of CD. IPSS has high sensitivity and specificity (95%) in differentiating CD from EAS but is an invasive test that is only available at specialized centers with significant experience/expertise. A combination of CRH and desmopressin test with a whole-body CT scan is proposed as an alternative to IPSS.^44^

Common causes of EAS are carcinoid tumor of the lung, pancreatic islet cell tumor, medullary carcinoma of the thyroid, small cell tumor of the lung, and thymic tumor. Whole-body CT scan (neck, chest, abdomen, and pelvis) is the imaging modality for anatomical localization of the EAS. Patients with negative anatomic imaging but with a strong clinical suspicion for EAS should undergo nuclear imaging (^68^Ga DOTATATE-and/ or FDG-PET).^40^

A high-dose dexamethasone suppression test has been used to differentiate between CD and EAS. This test used the theory that patients with CD retain the ability to suppress ACTH and cortisol production (with cortisol values <1.8 ug/dL or 50 nmol/L) in response to high-dose dexamethasone, whereas EAS patients don’t. However, some EAS patients, especially those with benign carcinoid tumors, retain the ability to suppress the ACTH in response to high-dose dexamethasone, lowering the discriminatory ability of high-dose suppression tests.^40^

Surgical resection of the culprit lesion, if feasible, offers the highest possibility of attaining a cure or prolonged remission. Hence, it is considered the preferred treatment. However, other modalities (radiotherapy or pharmacotherapy) may be required, either before (in those with severe pre-operative hypercortisolism), or after surgery (in those with persistent or recurrent disease) especially in patients with CD.^40^ Pharmacotherapeutics can be pituitary-directed drugs (pasireotide or cabergoline), adrenal steroidogenesis inhibitors (metyrapone, ketoconazole, levoketoconazole, etomidate, mitotane, osilodrostat), or glucocorticoid receptor antagonist (mifepristone). Bilateral adrenalectomy followed by adrenal steroid replacement can be considered in cases where restoration of rapid eucortisolism is needed, or where other treatments have failed or are inappropriate.^40^

**Table 4:** A proposed scoring system for the pretest probability of^48^ pheochromocytoma and paraganglioma

Clinical feature for the likelihood of pheochromocytoma and paraganglioma	Point score
Pallor	+1 point
Palpitations	+1 point
Heart rate ≥85 bpm	+1 point
Tremor	+1 point
Nausea	+1 point
BMI >30 kg/m^2^	-1 point
BMI <25 kg/m^2^	+1 point

*BMI = body mass index..*

Patients with morbid obesity, psychiatric illness, alcohol excess, polycystic ovary syndrome, pregnancy, uncontrolled diabetes mellitus, anorexia, malnutrition, excessive exercise, severe illness, surgery, high CBG states, or glucocorticoid resistance may exhibit CS clinical features (though the cutaneous and muscular features are rare) and abnormal screening tests (mildly elevated or equivocal).^40^ These are known as pseudo-CS, non-neoplastic hypercortisolism, or physiological cortisol hypersecretion. Dexamethasone-CRH test and/or desmopressin test can be used to rule out pseudo-CS.

## Pheochromocytoma and paraganglioma

Pheochromocytomas (PCCs) -neuroendocrine tumors that originate from the adrenal medulla -constitute around 80–85% of PPGL. Paragangliomas (PGLs) -neuroendocrine tumors that originate from the paravertebral sympathetic ganglia in the chest, abdomen, or pelvis, or the parasympathetic ganglia in the neck and skull base -constitute around 15–20% of PPGL.^45,46^ The PPGL has an incidence of 6.6 cases per million/year and a prevalence of 65 per 100,000 people. Among patients with an adrenal incidentaloma, pheochromocytoma accounts for around 7%, whereas in the general hypertensive population, PPGL is rare accounting for <1%. However, this diagnosis should not be missed due to the negative impact associated with a delayed or missed diagnosis.

Either germline mutation (in up to 40%) or somatic mutation (in around 30–40%) in one of the predisposition genes is identified in up to 70% of patients with PPGL.^47^ Mutations in the *VHL* (von Hippel Lindau), *RET*, and *NF1* (Neurofibromin 1) genes occur frequently in PCCs and rarely in PGLs. On the other hand, mutations in the *SDHx* group (succinate dehydrogenase) genes, chiefly *SDHB* and *SDHD*, are frequent in PGLs but rare in PCCs. PPGLs are classified into three major molecular clusters based on the mutations that are driving the pathogenesis. The cluster 1A includes *SDHx* mutations, cluster 1B includes *VHL* mutations, and cluster 2 includes *RET*, *NF1, TMEM127, and MAX* among many other mutations. The cluster 3 is less explored.

The classic PPGL triad including palpitation, headache, and diaphoresis are found in only 20% of patients. The most common sign found in up to 95% of PPGL patients is hypertension, which can be sustained (more with cluster 1 tumors that secrete norepinephrine; constitute 50% of PPGL), episodic or paroxysmal (more with cluster 2 tumors that secrete epinephrine; constitute 45% of PPGL). Normotension is also seen (more with cluster 1A -SDHx -tumors that secrete dopamine together with norepinephrine; constitute 5–15% of PPGL).^47^

PPGLs secreting predominantly or exclusively dopamine are very rare, and most occur with concomitant norepinephrine overproduction. In these patients, the vasodilatory effect of dopamine counteracts the vasoconstrictor effect of norepinephrine. Dopamine-secreting PGLs are mostly found incidentally on imaging or found because of pressure effects; these are generally larger, and often metastatic at diagnosis. Based on the clinical features, a scoring system has been proposed to establish the pretest likelihood of the disease, with a score ≥3 associated with a 5.8-fold pretest probability of PPGL^48^ (*Table 4*). The biochemical testing for PPGL using plasma free metanephrines or urinary fractionated metanephrines is indicated in patients with the features given in *Table 5*.^49^

**Table 5:** Recommendations for biochemical testing for pheochromocytoma and paraganglioma^49^

Clinical features of PPGL; spontaneous or provoked using medications or by other triggers
Cardiovascular events including Takotsubo cardiomyopathy with clinical features suggesting PPGL
Presence of adrenal incidentaloma of ≥10 HU density (with or without hypertension)
BMI <25 kg/m^2^ with type 2 diabetes mellitus ± clinical features of catecholamine excess
Surveillance screening: carrier of a germline mutation in one of the PPGL predisposition genes
Surveillance screening: syndromic features suggestive of genetic or syndromic PPGL
Surveillance screening: history or family history of PPGL

*BMI = body mass index; PPGL = pheochromocytoma and paraganglioma.*

Plasma free metanephrines and urinary fractionated metanephrines have very high negative predictive value (above 99%) and comparable specificity (around 94%) provided appropriate precautions (at least 20 min of supine rest, avoidance of strenuous exercise and potentially interfering medications sufficiently before testing) are taken. Thus, a negative test virtually rules out a PPGL except in those with very small tumors or in those with non-functional head and neck PGLs.^49^ In patients at low risk, both plasma and urine metanephrines have the same diagnostic accuracy. However, in those at high risk (adrenal incidentaloma and surveillance screening), plasma has superior diagnostic accuracy. Many medications are associated with falsely high metanephrines due to pharmacodynamic (antidepressants, MAO inhibitors, levodopa, sympathomimetics, phenoxybenzamine) or analytical (acetaminophen, sotalol) interference. The analytical interference is minimal for LC-MS/MS and hence it is the preferred method. As some foods can interfere with the measurement of 3-methoxytyramine (3MT), overnight fasting and avoidance of these foods are important for 3MT estimation.

Patients with a 2-fold rise of a single metabolite or simultaneous rise in 2 or more metabolites (metanephrine, normetanephrine, and/or 3MT) are less likely to be false-positive and hence can proceed with imaging. On the contrary, patients with <2 fold rise of a single metabolite without any potential for a false-positive result (including heart failure, OSA, inappropriate sampling, drug interference, and incorrect cut-off value) can undergo a clonidine suppression test if the pretest probability for PPGL was high. However, further tests are not needed for patients with <2 fold rise of a single metabolite with a low pretest probability for PPGL.^49^

Imaging studies should be done only after biochemical testing except in critically ill patients and/or patients in the intensive care setting, and when non-secretory tumors are suspected. Anatomical imaging by dedicated contrast-enhanced CT or MRI is enough to locate a PPGL and to plan the surgery.^50^ In anatomical imaging, PPGLs can be homogeneous or heterogeneous with necrosis, hemorrhage, cystic changes, and calcifications. In the representative area of PPGL, the unenhanced CT attenuation is almost invariably >10 HU in contrast to adrenal adenomas. A high-intensity T2-weighted signal is typical for PCC in MRI, but this has less sensitivity as it occurs in only one-third of tumors. When anatomical imaging is combined with functional imaging there is enhanced specificity and sensitivity for detection of metastases and multifocality. Functional imaging is a crucial investigation for those with a high risk of recurrence, metastatic or multifocal disease as this imaging can be linked with targeted radionuclide therapy. ^68^Ga-DOTA-SSA PET is the preferred functional imaging in metastatic PPGL, and it has a superior detection rate of 93% in comparison to 74% for ^18^F-FDG PET and 38% for ^123^I-MIBG scan in patients with PPGL.^45^ ^68^Ga-DOTA-SSA PET is mandatory for selecting patients for ^177^Lu-DOTATATE therapy. The optimal choice of a radiopharmaceutical for functional imaging depends on genotype, biochemical phenotype, location, and PPGL size.^50^

**Table 6:** Summary of clinical characteristics of various adrenal causes of endocrine hypertension

Endocrine HTN	Inheritance	Gene	Clinical presentations	Treatment
Low renin -high aldosterone monogenic hypertension with hypokalaemia and metabolic alkalosis				
Familial hyperaldosteronism				
FH-l orGRA	AD	*CYP11B1/CYP11B2*	Early-onset severe hyperaldosteronism	Glucocorticoids or MRA
FH-I I	AD	*CLCN2*	Early hyperaldosteronism, variable presentation	MRA
FH-I I 1	AD	*KCNJ5*	Early severe hyperaldosteronism-massive BAH	MRA +/-adrenalectomy
FH-I V	AD	*CACNA1H*	Early hyperaldosteronism, developmental dela/	MRA +/-CCBs
PASNA	AD	*CACNA1D*	Early severe hyperaldosteronism, seizure, neurologic illness	MRA +/-CCBS
APA	Somatic	*CLCN2, KCNJ5, CACNA1H, CACNA1D, ATP1A1, ATP2B3, CTNNB1, PRKACA*	Spontaneous or diuretic-induced hypokalaemia, resistant HTN, OSA, AF, CVD	MRA +/-adrenalectomy
	Germ l ine	*ARMC5*	Hypokalaemia, resistant HTN, OSA, AF, CVD	MRA +/-adrenalectomy
Low renin -low aldosterone monogenic hypertension with hypokalaemia and metabolic alkalosis				
AME	AR	*HSD11B2*	IUGR, failure to thrive, severe HTN; NC-AME: miId HTN with onset in adults	MRA, thiazide, low sat diet
11ß-OHase deficiency CAH	AR	*CYP11B1*	Early HTN, virilisation (46 XX) and raised 17-OHP	Glucocorticoid for all types of CAH
17α-OHase deficiency CAH	AR	*CYP17A1*	Early HTN, ambigucus genitalia(46XY), low 17-OHP	Sex hormone for 17a-OHase deficiency
POR deficiency CAH	AR	*POR*	Early HTN, ambiguous genitalia(46XY), low 17-OHP	Amiloride, spironolactone or CCB for HTN control
Liddle syndrome	AD	*SCNN1B, SCNN1G, SCNN1A*	Early-onset severe HTN, fails to respond to MRAs	Amiloride and low salt diet
Geler syndrome	AD	*NR3C2*	Early-onset severe HTN exacerbated by pregnancy and MRA	Amiloride and low salt diet
Chrousos syndrome	AD	*NR3C1*	High cortisol and ACTH without Cushing features	High dose Glucocorticoids
Cushing syndrome			Striae, easy bruising, facial plethora, proximal muscle atrophy, osteoporosis	Surgery +/-anti HT drugs
Genetic forms of Cushing syndrome				
MEN1	AD	*MEN1*	Parathyroid, pancreas and pituitary (rarely adrenal) tumors	Surgery +/-medical therapy of Cushing
MEN4	AD	*CDKN1B*	Similar clinical phenotype to MEN1, but with different mutation	Surgery +/-medical therapy of Cushing
PPNAD	AD	*PRKAR1A*	Component of Carney complec	Bilateral adrenalectomy
Carney	AD	*PRKAR1A*	Spotty skin pigmentation, myxomatous lesions, endocrine tumors	Surgery +/-medical therapy of Cushing
MAS	Somatic	*GNAS*	Cafe-au-l ait spots, fibrous dysplasia and endocrine excess syndromes	Surgery +/-medical therapy of Cushing
FIPA	AD	*AIP*	Familial pituitary tumors with negative MEN1 mutations	Surgery +/-medical therapy of Cushing
PBMAH	AD	*ARMC5*	80% of familial PBMAH, bilateral disease, adrenal Cushing	Bilateral adrenalectomy
PBMAH	Somatic	*KDM1A*	Food-dependent Cushing syndrome with ectopic expression of GIP-R	Bi lateral adrenalectomy
LFS	AD	*TP53*	Sarcoma, breast, leukaemia, and adrenal gland cancer syndrome	Surgery +/-medical therapy of Cushing
FAP	AD	*APC*	Colon polyp, thyroid/pancreas cancers, desmoid tumors, PBMAH	Surgery +/-medical therapy of Cushing
High renin -high aldosterone monogenic hypertension with hypokalaemia and metabolic alkalosis				
JGCT	Polygenic	*p53* and *Rb* deletions	Adolescent or young adult, mostly women-refractory HTN	Surgery +/-Aliskiren
Low renin normal aldosterone monogenic hypertension with hyperkalaemia and metabolic acidosis				
Gordon or FHHt	AD	*WNK4, WNK1, KLHL3, CUL3*	HTN, hyperkalemia and type 4 RTA in patients with preserved renal function	Thiazide and low salt diet
Normal renin -normal aldosterone monogenic hypertension with normokalaemia and normal acid-base balance				
PPGL			HTN; sustained/episodic, headache, palpitation, disphoresis	Surgery +/-anti HT drugs
Familial forms of PPGL				
SDH related PPGL	AD	*SDHA, SDHB, SDHC, SDHD, SDHAF2*	Head and neck paraganglioma, pheochromocytoma	Surgery, chemotherapy, PRRT, anti-VEGF therapy
MEN2	AD	*RET*	PHPT, medullary thyroid cancer and pheochromocytoma	Surgery +/-chemotherapy +/-PRRT
VHL syndrome	AD	*VHL*	Pheochromocytoma, paraganglioma, hemangioblastoma	Surgery +/-targeted therapy with Belzutifan
NF type 1	AD	*NF1*	Pheochromocytoma, neurofibroma, Lisch nodule, freckling	Surgery +/-chemotherapy +/-PRRT
MAX related PPGL	AD	*MAX*	Pheochromocytoma, paraganglioma, and renal cell cancer	Surgery +/-chemotherapy +/-PRRT
TMEM127-related PPGL	AD	*TMEM127*	Pheochromocytoma, paraganglioma, and renal cell cancer	Surgery +/-chemotherapy +/-PRRT
Pacak-Zhuang syndrome	Somatic	*EPAS1*	Females; polycythemia, PPGL & duodenal somatostatinoma	Surgery +/-targeted therapy with Belzutifan
HTNB (Bilginturan syndrome)	AD	*PDE3A*	Early-onset severe HTN, short stature and brachydactyly	

*ACTH = Adrenocorticotrophin; AD = Autosomal Dominant; AME = Apparent Mineralocorticoid Excess; APA = Aldosterone-Producing Adenoma; AR = Autosomal Recessive; CAH = Congenital Adrenal Hyperplasia; CCB = Calcium Channel Blocker; ENaC = Epithelial Sodium Channel ; FAP = Familial Adenomatous Polyposis; FH = Familial Hyperaldosteronism; FHHt = Familial Hyperkalemic Hypertension; FIPA = Familial Isolated Pituitary Adenoma; GIP-R = Glucose-Dependent Insulinotropic Polypeptide Receptor; GRA = Glucocorticoid Remediable Aldosteronism ; HTN = Hypertension; HTNB = hypertension and Brachydactyly Syndrome (Bilginturan syndrome); IUGR = Intrauterine Growth Restriction; JGCT = JuxtaGlomerular Cell Tumor; LFS = Li-Fraumeni Syndrome; MAS = McCune-Albright syndrome; MEN = Multiple Endocrine Neoplasia; MRA = Mineralocorticoid Receptor Antagonist; NC-AME = Nonclassic Apparent Mineralocorticoid Excess; NF = Neurofibromatosis; PASNA = Primary Aldosteronism, Seizures, and Neurological Abnormalities; PBMAH = Primary bilateral macronodular adrenal hyperplasia; PHPT = Primary hyperparathyroidism; POR = Cytochrome P450 oxidoreductase; PPGL = Pheochromocytoma Paraganglioma; PPNAD = Primary Pigmented Nodular Adrenocortical Disease; RTA = Renal Tubular Acidosis; SDH = Succinate Dehydrogenase; VHL = Von Hippel Lindau.*

Laparoscopic or open resection is the only therapy with curative intent in patients with PPGL. Systemic therapies including chemotherapy, targeted therapy with tyrosine kinase inhibitors, and radionuclide therapy are alternative options for inoperable or metastatic disease.^51,52^ Manipulation during surgery can induce a catecholamine surge causing hypertensive crisis, myocardial infarction, cardiac arrhythmia, and acute pulmonary edema.^45,46^ Moreover, severe, and refractory hypotension can develop from sudden catecholamine withdrawal after PPGL resection. Hence, adequate preoperative preparation is needed even in normotensive PPGL patients with normal metanephrine levels before surgery. Liberal fluid and salt intake are advised in the pre-operative period to restore the catecholamine excess mediated volume contraction, which would lessen the volume expansion that occurs after PPGL removal. Preoperative targets are seated blood pressure <130/80 mmHg, upright systolic blood pressure >90 mmHg, seated heart rate of 60–70 bpm, and an upright heart rate of 70–80 bpm.^45,46^

α-blockers should be started at least 7–14 days before surgery with gradual dose escalation until expected targets are reached.^45,46^ Choices include phenoxybenzamine, a nonselective and noncompetitive α-blocker, or doxazosin, a selective and competitive α1-adrenergic blocker. There is no demonstrable superiority of one over another, though phenoxybenzamine was associated with better per-operative hemodynamic stability, but without any better clinical outcomes. After achieving adequate α-blockade (usually after 3–5 days), the β-blockade can be started, to improve blood pressure control and to minimize the α-blockade induced undesirable tachycardia. β-blocker should never be used as monotherapy as this would cause unopposed α-adrenoceptor stimulation causing pheochromocytoma crisis. Calcium channel blockers may be added to optimize blood pressure control.^45,46^ In some difficult PPGL resection or PPGL cases with anticipated large catecholamine release (e.g., chemotherapy), metyrosine, a tyrosine hydroxylase inhibitor may be used. Sadly, metyrosine is not available in many countries and is very costly. α-blockade is not indicated in dopamine-secreting PPGLs because of severe hypotension risk.

Several surrogate markers are used to determine the metastatic potential of PPGLs which contribute to various scores including the Pheochromocytoma of the Adrenal Gland Scaled Score (PASS), the Grading of Adrenal Pheochromocytoma and Paraganglioma (GAPP), and the Composite Pheochromocytoma-paraganglioma Prognostic Score (COPPS).^45,46^ Both PASS and GAPP systems are used globally to assess the malignant potential of PCCs. PASS score of 4 or more is highly sensitive for malignant behavior (100% sensitivity and 75% specificity), whereas a tumor with a GAPP score of <3 has a metastatic rate of 3% and a 5 year survival of 100%.^45,46^

**Figure 3:**
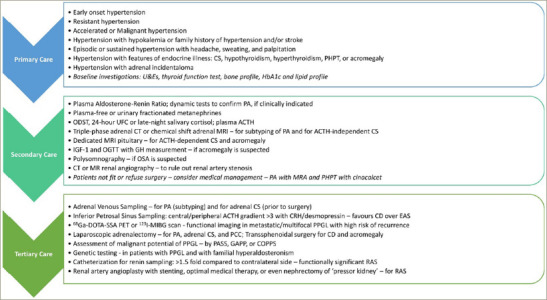
Algorithm for screening and work up of patients with suspected endocrine hypertension at primary, secondary and tertiary-care levels

All subjects with PCCs and PGLs should be involved in the discussion about genetic testing, irrespective of age or tumor location.^53^ Although clinical and biochemical features can suggest a specific causal gene, they do not always predict the underlying genotype. The genetic testing should ideally be performed using the targeted next-generation sequencing (NGS) which would enable testing of several genes in one panel from the DNA acquired either from the peripheral blood, the buccal swab, or, more recently, from the tumor tissue. The latter enables the detection of not only the germline mutations found in the tumorous and peripheral DNA but also the somatic mutations found only in tumorous DNA.

## Other endocrine causes of hypertension

*Table 6* provides an overview of various other adrenal causes of endocrine hypertension with special attention to inheritance, genetic mutation, clinical features, and treatment.^54–59^ Chronic ingestion of licorice can cause apparent mineralocorticoid excess syndrome. Co-secretion of aldosterone and cortisol from an adrenal tumour can lead to the development of Connshing (CoSh) syndrome.^60^ ACTH dependent and independent CS can rarely co-exist.^61^ Though the common components of Multiple Endocrine Neoplasia 2 (MEN2) are primary hyperparathyroidism, PCC, and medullary thyroid cancer, it can rarely cause ACTH dependent CS (both Cushing’ disease and ectopic ACTH or CRH secretion).^62^ Finally, ACTH production by some PCCs can cause ACTH independent CS via a paracrine action on the ipsilateral adrenal cortex causing subclinical or even clinical CS.^63^ The principles that we have discussed in this article can also be applied in the investigation and management of patients with adrenal incidentaloma. Adrenal lesions from PCC are often larger in comparison to PA or CS. Apart from the adrenal causes of hypertension discussed, many other endocrine pathologies can present with hypertension including growth hormone excess and deficiency.^64^ hyperparathyroidism, thyroid hormone excess or deficiency,^65^ and OSA.^66^ We have not discussed them here as other recent literature elaborates these conditions in detail.^11,20^

*Figure 3* shows an algorithm for screening and work up of patients with suspected endocrine causes of hypertension at primary, secondary and tertiary-care levels.

## Conclusion

Although endocrine hypertension is common with a prevalence of about 10% among hypertensive individuals and about 20% of those with resistant hypertension, the diagnosis is often missed because of poor awareness among healthcare workers and the subtle symptoms in individuals with the disease. Prompt early diagnosis and appropriate management often result in a cure of the disease or at least better control of hypertension and the likelihood of end-organ complications. As some of these disorders are inherited, proper genetic testing with methods such as NGS can optimize the care of patients with the disease. There is an urgent need to increase global awareness among medical professionals to improve the diagnosis and management of endocrine hypertension.
